# Replacement of pr gene with Japanese encephalitis virus pr using reverse genetics reduces antibody-dependent enhancement of dengue virus 2 infection

**DOI:** 10.1007/s00253-015-6819-3

**Published:** 2015-07-29

**Authors:** Ying Wang, Lulu Si, Yayan Luo, Xiaolan Guo, Junmei Zhou, Danyun Fang, Huijun Yan, Gucheng Zeng, Lifang Jiang

**Affiliations:** Key laboratory for Tropic Diseases Control, Ministry of Education of China, Department of Microbiology, Zhongshan School of Medicine, Sun Yat-sen University, Guangzhou, Guangdong 510080 China

**Keywords:** Dengue virus, ADE, Infectious cDNA clone, Chimeric virus, prM antibodies

## Abstract

Severe dengue is more likely found during secondary heterologous dengue virus (DENV) infection or primary infection of infants born to dengue-immune mothers and led to the hypothesis of antibody-dependent enhancement (ADE). It has been reported that pre-membrane (prM)-reactive antibodies do not efficiently neutralize DENV infection but instead potently promote ADE infection. Meanwhile, these enhancing anti-prM antibodies mainly react with the precursor (pr) peptide. To evaluate the effect of pr gene substitution on neutralization and ADE of DENV infection, a novel chimeric dengue virus (JEVpr/DENV2) was rationally constructed by replacing the DENV pr gene with Japanese encephalitis virus (JEV) pr gene, based on the full-length infectious complementary DNA (cDNA) clone of DENV2 ZS01/01. We found that chimeric JEVpr/DENV2 showed reduced virulence and good immunogenicity. In addition, anti-JEVpr/DENV2 sera showed broad cross-reactivity and efficient neutralizing activity with all four DENV serotypes and immature DENV2 (ImDENV2). Most importantly, compared with anti-DENV2 sera, anti-JEVpr/DENV2 sera showed significantly reduced enhancing activity of DENV infection in K562 cells. These results suggest that the ADE activities could be reduced by replacing the DENV pr gene with JEV pr gene. These findings may help us better understand the pathogenesis of DENV infection and provide a reference for the development of a vaccine against DENV.

## Introduction

Currently, the four serotypes of dengue virus (DENV) are the major cause of the most prevalent arthropod-borne viral infection worldwide. A recent study estimated that approximately 390 million individuals are infected with DENV globally per year, of which 96 million are clinically apparent cases (Bhatt et al. 2013). This infection total is more than three times the number of cases estimated by the WHO. DENV infection can result in asymptomatic infection or self-limited dengue fever (DF); however, an increasing number of people are at a risk of progressive development to more severe and potentially fatal clinical manifestations including dengue hemorrhagic fever (DHF) and dengue shock syndrome (DSS) (Halstead 2007; W.H.O. 2009). There are currently no effective commercial antiviral therapeutics to treat DENV infection, only supportive treatment.

Infection with one serotype stimulates an effective and possibly life-long immunity against the reinfection with the same serotype; however, it does not protect against other serotypes (Rothman 2004). Several retrospective and prospective studies have revealed that secondary infection with different DENV serotypes increases the risk of developing severe dengue symptoms (Halstead et al. 1970; Thein et al. 1997; Vaughn et al. 2000). In addition, infants born to dengue-immune mothers are at risk of developing more severe dengue symptoms during primary infection (Halstead et al. 2002; Kliks et al. 1988; Simmons et al. 2007). Antibody-dependent enhancement (ADE) has been proposed as the most compelling explanation for severe dengue (Halstead and O’Rourke 1977; Kliks et al. 1989). According to this hypothesis, non-neutralizing antibodies or cross-reactive antibodies at sub-neutralizing concentrations can enhance DENV infection by targeting the virus to Fc receptor (FcR)-bearing cells and hence may contribute to an increased dengue-infected cell mass, eventually causing a higher virus load (Halstead 1970, 2003; Kliks et al. 1988). The difficulty of balancing immunity to the four serotypes and the risk of ADE are major hurdles in the development of a tetravalent vaccine against DENV (Whitehead et al. 2007).

DENV contains a 10.7-kb positive-sense ssRNA genome. It encodes three structural proteins (capsid, C; pre-membrane, prM; and envelope, E) and seven non-structural (NS) proteins (NS1, NS2a, NS2b, NS3, NS4a, NS4b, and NS5). The prM protein is a 166-amino acid protein, which is believed to acts as a chaperone for the folding and assembly of E protein and to prevent the premature fusion of the virus to membranes inside the producing cell (Lorenz et al. 2002). prM can be cleaved into a C-terminal M portion, which remains associated with the virus particle. The other is an N-terminal 91-amino acid precursor (pr) peptide that dissociates upon release from the virion (Yu et al. 2008) by the cellular endoprotease furin, leading to the formation of mature infectious virions. The cleavage of prM protein is required for the activation of DENV infectivity. Interestingly, there are as many as 30 % prM-containing immature particles in virus preparations released from both BHK-21 and C6/36 cells (Junjhon et al. 2008; Zybert et al. 2008), indicating that the prM cleavage and maturation of dengue virus are inefficient. Thus, cells infected with DENV secrete a heterogeneous mixture ranging from fully mature (containing M) and partially mature (containing prM and M) to fully immature (containing prM) virions (Cherrier et al. 2009; Junjhon et al. 2010; Zybert et al. 2008). It has been proven that fully immature virus particles are normally non-infectious whereas fully mature and partially mature virus particles are infectious (Dejnirattisai et al. 2010; Rodenhuis-Zybert et al. 2010, 2011b; Zybert et al. 2008). Additionally, the maturation state of virus particles can influence the capacity of antibodies to neutralize and enhance DENV infection (Nelson et al. 2008; Rodenhuis-Zybert et al. 2010, 2011b).

Numerous studies have reported that anti-prM antibodies are commonly found in the sera of dengue-infected patients (Bray and Lai 1991; Cardosa et al. 2002; Se-Thoe et al. 1999) and the levels of prM antibodies were significantly higher in patients with secondary infection than sera from primary DENV infection (Lai et al. 2008), suggesting prM-specific antibodies play critical roles in human immune responses to DENV infection in both primary and secondary infection. Additionally, a previous study found a positive correlation between the circulating prM antibody level and disease severity (Rai et al. 2008). Furthermore, many findings demonstrated that prM antibodies are highly cross-reactive among the four DENV serotypes and do not efficiently neutralize infection, but potently promote ADE even at high concentrations (Beltramello et al. 2010; Dejnirattisai et al. 2010; Huang et al. 2006). Another study in infants has also implicated the susceptibility of infants to severe dengue in the presence of anti-prM antibodies (Chau et al. 2009). Most importantly, both mouse and human prM-specific antibodies were shown to render essentially non-infectious immature DENV highly infectious and nearly as infectious as the wild-type virus (Chan et al. 2012; Dejnirattisai et al. 2010; Rodenhuis-Zybert et al. 2010). Taken together, these studies suggest that prM-specific antibodies may play an important role in enhancing DENV infection in humans.

Another previous study reported that these anti-prM antibodies mainly (at least five of six) reacted with the pr peptide (Dejnirattisai et al. 2010). In addition, it has been reported that anti-prM monoclonal antibody 4D10, pr4, 2H2, and 70–21 could enhance DENV infectivity. The specific epitopes of 4D10, pr4, 2H2, and 70–21 were mapped to amino acid residues 14–18, 19–34, 40–49, and 53–67 of pr peptide, respectively (Falconar 1999; Huang et al. 2008; Luo et al. 2015, 2013). Taken together, these findings indicated the critical role of pr peptide in ADE of DENV infection. Therefore, we hypothesize that replacing the DENV pr gene with Japanese encephalitis virus (JEV) pr could minimize the anti-pr response and further reduce the ADE mediated by anti-pr antibodies. To investigate whether the replacement of the DENV pr gene with JEV pr gene would reduce or even ablate the ADE of DENV infection, we designed and constructed chimeric JEVpr/DENV-2 (substitution of DENV pr gene with JEV pr gene) using reverse genetics. The results showed that the chimeric JEVpr/DENV2 is attenuated and immunogenic. The anti-JEVpr/DENV2 sera showed broad cross-reactivity and efficient neutralizing activities with all four DENV serotypes and immature DENV2 (ImDENV2) as anti-DENV2 does. We also found that the ADE mediated by anti-JEVpr/DENV2 sera was significantly reduced, as compared with anti-DENV2 sera. These findings may have implications for DENV pathogenesis as well as for the development of DENV vaccines.

## Materials and methods

### Cells, viruses, and plasmid

C6/36, a cell line derived from the mosquito *Aedes albopictus* was cultured in Modified Essential Medium (MEM) supplemented with 10 % heat-inactivated fetal bovine serum (FBS), 100 U/ml penicillin, 100 μg/ml streptomycin, and 2 mM l-glutamine at 28 °C. Baby hamster kidney-21 (BHK-21) cells derived from the kidney of *Mesocricetus auratus* and human adenocarcinoma LoVo cells derived from left supraclavicular region metastasis were cultured in Dulbecco’s Modified Eagle’s Medium (DMEM) supplemented with 10 % FBS, 100 U/ml penicillin, 100 μg/ml streptomycin, and 2 mM l-glutamine at 37 °C, 5 % CO_2_. Human erythroleukemic K562 cells derived from the bone marrow were maintained in Iscove’s Modified Dulbecco’s Medium (IMDM) supplemented with 10 % FBS, 100 U/ml penicillin, 100 μg/ml streptomycin, and 2 mM l-glutamine at 37 °C, 5 % CO_2_. All cells were purchased from ATCC. All cell culture-related reagents were obtained from GIBCO.

The DENV2 strain ZS01/01 (GenBank accession no. EF051521.1) has been previously isolated, sequence-verified, and preserved in our laboratory. DENV1 strain Hawaii (GenBank accession no. EU848545), DENV3 strain H87 (GenBank accession no. M93130), DENV4 strain H241 (GenBank accession no. AY947539), and JEV (GenBank accession no. JN711458.1) were supplied by the Institute for Viral Disease Control and Prevention, China CDC. The recovered virus derived from the complementary DNA (cDNA) clone of DENV2 strain ZS01/01 (pACYC177-DENV2) is termed RecDENV2 (GenBank accession no. KR920365). All viruses were propagated on C6/36 cells. Briefly, monolayer of C6/36 cells was infected at a multiplicity of infection (MOI) of 1. The virus supernatants were harvested at 72 h post-infection (hpi), cleared from cellular debris by low-speed centrifugation (1000×*g*, 5 min), aliquoted, and stored at −80 °C. Fully ImDENV2 particles were produced on furin-deficient LoVo cells as described before (Zybert et al. 2008). Briefly, LoVo cells were infected with DENV2 strain ZS01/01 at an MOI of 10 for 1.5 h at 37 °C. Viral inoculum was removed and fresh medium was added after washing the cells twice with phosphate buffer solution (PBS). At 72 hpi, the supernatant containing the virus particles was harvested, cleared from cellular debris by low-speed centrifugation (1000×g, 5 min), and stored in aliquots at −80 °C. Virus titers were determined by standard plaque assay on BHK-21 cells.

The vector pACYC177 (GenBank accession no. X06402) used in this study was kindly provided by New England Biolabs.

### Antibodies and sera

Monoclonal antibodies specific for DENV prM (2H2, IgG2a) and *flavivirus* E protein (4G2, IgG2a) were produced by intraperitoneal inoculation of BALB/c mice with hybridoma purchased from ATCC. Antisera of DENV2 and JEVpr/DENV2 were produced by subcutaneous (sc) injection of BALB/c mice with inactivated virus, individually.

### Plaque-forming assay

BHK-21 cells were plated at a density of 1 × 10^5^ cells per well in 24-well plates containing 500 μl of medium and cultured overnight at 37 °C. Then, 250 μl of serially 10-fold diluted virus solution was added to each well and incubated at 37 °C for 2 h with intermittent manual shaking every 20 min. The virus was removed and 1 ml of DMEM medium containing 2 % FBS, 100 U/ml penicillin, 100 μg/ml streptomycin, 2 mM l-glutamine, and 1.2 % (*w*/*v*) carboxymethylcellulose (Sigma) was added, and plates were incubated further at 37 °C for 7 days. The cells were fixed with 4 % (*v*/*v*) formaldehyde and the plaques were visualized by staining with 0.8 % (*w*/*v*) crystal violet. Virus concentrations were expressed as plaque-forming units per milliliter (PFU/ml).

### Construction of a full-length cDNA infectious clone of DENV2 strain ZS01/01

A reverse genetics system for the DENV2 strain ZS01/01 was firstly constructed. Briefly, based on the published nucleotide sequence of the virus strain, the full-length (10,723 bp) cDNA of DENV2 ZS01/01 were divided into four overlapping fragments and were amplified by PCR using Phusion® High-Fidelity DNA Polymerases (NEB). The primers (F1–F4) are listed in Table 1. A 15-bp overlap was included on each primer corresponding to upstream and downstream bases of the destination vector to enable ligation using In-Fusion™ HD Cloning Kit (Clontech). Restriction sites *Sac*II and the promoter (5′-ATTTAGGTGACACTATAG-3′) for SP6 RNA polymerase were engineered at the 5′ terminus of the cDNA. No extraneous nucleotide (nt) was inserted between SP6 promoter and the genomic cDNA. Unique *Cla*I was engineered at the 3′ terminus of the genomic cDNA to permit linearization prior to RNA transcription. F1 and F2 fragments (containing 1–5505 bp) were cloned into the *Sac*II/*Cla*I sites of pACYC177 first. Then, the plasmid containing F1 and F2 was digested with *Xho*I and *Cla*Iso that F3 and F4 fragments (containing 5426–10,723 bp) could be introduced to generate full-genome cDNA clone, named pACYC177-DENV2. In all cases, pACYC177 was used as plasmid vector and all clones were propagated in *Escherichiacoli* DH5α-competent cells (TaKaRa).

**Table 1:** Oligonucleotide primers used to construct the full-length cDNA clone of DENV-2 and JEVpr/DENV-2

Primer	Range (nt)	Length (bp)	Primer sequence (5′–3′)	Introduced sequence/restriction site
F1-f M1-f	1~25	2913	GAAAAACGGCTTTGCCGCGG **ATTTAGGTGACACTATAG**AGTTGTTAGTCTACGTGGACCGACA	SP6 promoter (bold) *Sac*II (underlined)
F1-r	2913~2884		GGTAAATACTCCAAAGCCATAGTCTTCAAC	
F2-f	2899~2926	2608	TTTGGAGTATTTACCACCAACATATGGC	
F2-r	5505~5481		CTTCCCATACAATCGATGCTCTGAGGAAATGGGTCTCTGCTT	*Cla*I (underlined)
F3-f	5416~5440	2312	TACATTTCAACTCGAGTTGAGATGG	*Xho*I (underlined)
F3-r	7727~7706		TGATGGTCCGTTTCTCCTCTTT	
F4-f	7712~7733	3012	GAGAAACGGACCATCACGCTGT	
F4-r	10,723~10,701		CTTCCCATACAATCGATAGAACCTGTTGATTCAACAGCAC	*Cla*I (underlined)
M1-r	446–417	484	AACTTCATCGCCACCACTGTTGGAATCATC	
M2-f	431–460	509	TGGTGGCGATGAAGTTGTCAAATTTCCAGG	
M2-r	939–913		TGTCATTGAAGGAGCGACAGCTGTCAG	
M3-f	924–949	1462	TCCTTCAATGACAATGCGTTGTATAG	
M3-r	2385–2360		CCCCACTAATACTAGTGACACAGACA	*Spe*I (underlined)

### Construction of chimeric JEVpr/DENV2

Based on pACYC177-DENV2, the DENV pr gene (439–711 bp) was replaced with that of JEV (477–752 bp) to construct the full-length cDNA clone of the chimeric JEVpr/DENV2 (named JEVpr/DENV2). Three fragments containing genome nt regions 1–446 (M1), 431–939 (M2), and 924–2385 (M3) were amplified by PCR using Phusion® High-Fidelity DNA Polymerases (NEB). M1 and M3 were amplified using the cDNA of DENV2 as the template, and M2 was amplified using the plasmid pET30a-JEVpr/DENV-M containing both JEVpr and DENV-M genes, which we constructed in another study. The sequences for forward and reverse primers are listed in Table 1. The plasmid of pACYC177-DENV2 was cut with *Sac* II and *Spe* I and ligated with M1, M2, and M3 using In-Fusion™ HD Cloning Kit (Clontech).

### In vitro transcription and transfection

Plasmid pACYC177-DENV-2 or pACYC177-JEVpr/DENV2 was linearized with *Cla*I prior to SP6 RNA polymerase transcription to produce RNA of defined length in the presence of an m^7^G (5′) ppp (5′) G cap analog. In vitro transcription was done using the RiboMAX Large Scale Production System (Promega) according to the manufacturer’s instructions. After removing the DNA template by digestion with DNase, the RNA concentration was quantitated by ultraviolet light absorbance and the integrity was visualized by gel electrophoresis. RNA transcripts (2 μg) were transfected into BHK-21 cells with 6 μl of Lipofectamine 2000 (Invitrogen) in 1 ml of serum-free medium. The mixture was then added to BHK-21 cells in six-well plates and incubated at 37 °C for 4 h. The Lipofectamine-RNA mixture was removed, and DMEM maintenance medium containing 2 % FBS was added. The transfected cultures were incubated for 3~5 days at 37 °C; the supernatants were then harvested and clarified by low-speed centrifugation. Clone-derived viruses were amplified in C6/36 cells for five passages and stored in aliquots at −80 °C. Virus titers were determined by standard plaque assay on BHK-21 cells.

### Nucleotide sequencing

Viral RNA was extracted using RNeasy mini kit (Qiagen), and a poly(A) tail was added to 3′ terminus of the RNA using Poly(A) Polymerase (TaKaRa) prior to cDNA synthesis. Then RACE Ready first-strand cDNA was synthesized using SMARTer™ RACE cDNA Amplification Kit (Clontech) according to the user’s manual. The whole genome including the 5′ and 3′ termini were amplified by PCR and cloned to pMD18-T vector (TaKaRa). Sequenced fragments were analyzed using DNASTAR software.

### Growth curves

Growth curves were done by infecting C6/36 and BHK-21 cells in T-12.5 flasks with parental DENV2 strain ZS01/01 or with clone-derived virus at an MOI of 0.01. The inoculum was removed after 1 h, and new maintenance medium containing 2 % FBS was added. The supernatant of infected cells was collected daily and stored at −80 °C. The titers of DENV2 in each collected sample were determined by plaque-forming assay on BHK-21 cells.

### Enzyme linked immunosorbent assay

To test the immunogenicity of JEVpr/DENV2, 96-well enzyme linked immunosorbent assay (ELISA) plates were coated with 100 μl inactivated JEVpr/DENV2 in carbonate/bicarbonate buffer at pH 9.6 per well overnight at 4 °C and blocked with 1 % bovine serum albumin (BSA) at 37 °C for 2 h. The plates were washed five times with PBST (0.5 % Tween-20 in PBS), then 1:100 diluted test or negative serum in PBST were added and incubated at 37 °C for 2 h. The plates were washed five times with PBST, and then incubated with 1:5000 dilution of horseradish peroxidase (HRP)-conjugated anti-mouse antibody (Abcam) at 37 °C for 1 h. After the final wash, HRP/substrate solution was added to each well and incubated at room temperature for 30 min. The reaction was stopped with 2 N H_2_SO_4_ and read at 450 nm in an ELISA Reader (Bio-Rad). A positive titer was defined as producing at least double the background absorbance.

### Indirect immunofluorescence assay

C6/36 or BHK-21 cells grown in 24-well plates containing a coverslip inside were infected with viruses at an MOI of 0.01 for 48 h. Then, the coverslips containing infected cells were fixed in 4 % paraformaldehyde for 20 min and permeabilized with 0.5 % Triton X-100 in PBS. The coverslips were blocked with 5 % BSA. Each coverslip was incubated with a 1:1000 dilution of primary antibody (anti-DENV prM antibody (2H2) or anti-E antibody) for 1 h at 37 °C and rinsed three times in PBS. Samples were then incubated with a 200-fold dilution of Alexa Fluor 488-conjugated goat anti-mouse IgG (Invitrogen) for 30 min at 37 °C and washed four times in PBS. The coverslips were fixed to glass slides and nuclei were stained with DAPI (Sigma). Images were acquired using a fluorescent microscope (Zeiss).

### Neutralization assay

The neutralizing activity of antisera was measured by a constant virus-serum dilution 50 % plaque reduction neutralization test (PRNT_50_). Briefly, serial twofold dilutions of inactivated sera (starting at 1:10) were mixed with an equal volume of approximately 30 PFU DENV1-4 and ImDENV2 in DMEM containing 2 % FBS for 2 h at 37 °C. The normal mouse serum at 1:10 dilution was used as the control. The virus-serum mixtures were then transferred to BHK-21 cell monolayers in 24-well plates followed by the plaque-forming assay as described above. The percentage of plaque reduction was calculated as previously described (Lok et al. 2008). The serum titer required to reduce dengue viral plaques by 50 % (50 % neutralization titer) was calculated from three experiments with the Reed-Muench method.

### Antibody-dependent enhancement assay

The antibody-dependent infection enhancement of antisera was detected by flow cytometry as described previously (Beltramello et al. 2010; Dejnirattisai et al. 2010; Huang et al. 2006; Luo et al. 2013). Briefly, twofold serially diluted serum (starting at 1:10) was incubated with an equal volume of DENV1-4 and ImDENV2 for 1 h at 37 °C, and then the virus-serum complexes were transferred to K562 cell suspension (2 × 10^5^/sample) at an MOI of 1 and incubated at 37 °C for 3 days. The cells were assayed for virus infection by flow cytometric analysis.

For dengue virion binding, infected K562 cells were fixed and permeabilized with Cytofix/Cytoperm™ Fixation/Permeabilization kit (BD) according to the manufacturer’s recommendation. DENV antigens were then stained intracellularly with specific anti-E antibody (4G2) followed by FITC-conjugated goat anti-mouse IgG antibody (Santa Cruz). The samples were washed twice and percentage of K562 cells infected by DENV was detected by flow cytometry.

### Mouse experiments

Research involving animals was approved by and carried out in strict accordance with the guidelines of the Animal Experimentation Ethics Committee of Sun Yat-sen University with two ethical approval numbers of 2013-004 and 2013-005. Animals were purchased from the Center of Experimental Animal of the Sun Yat-sen University.

#### Neurovirulence in suckling mice

For neurovirulence tests, groups of 3-day-old Kunming (Km) mice (*n* = 8–12 mice/group) were inoculated by the intracerebral (ic) route with 10-fold dilutions of RecDENV2 or its parental DENV2, 4000 or 800 PFU JEVpr/DENV2, or PBS, respectively. Animals were monitored for 21 days after inoculation. Mice found in a moribund condition were euthanatized and scored as dead. Virus doses inducing a 50 % mortality rate (LD_50_) were calculated using the method of Reed and Muench.

#### Immunization and protection assay in adult BALB/c mice

To assess the immunogenicity of the chimeric JEVpr/DENV2, groups of 4-week-old female BALB/c mice (*n* = 20 mice/group) were immunized with 100 μg inactivated chimeric JEVpr/DENV2 or PBS by the sc route, each group comprising 20 mice. The immunogen was emulsified with complete Freund’s adjuvant (Sigma) for the first immunization. Mice were then immunized with the immunogen emulsified with incomplete Freund’s adjuvant (Sigma) at the same dose at week 2 and 4. The serum was collected by tail vein puncture at 2, 4, and 6 weeks after immunization and stored at 20 °C. Antibody titer was measured by ELISA, and serum before immunization at 1:100 dilution was used as the background.

To test the protection response in adult BALB/c mice, 2 weeks after the last immunization, groups of mice (*n* = 5 mice/group) were injected by peritoneal injection of DENV1-4 (10^6^ PFU/mouse), separately. The viral RNA copy numbers of sera were quantified at 6, 12, 24, 48, and 72 h by qRT-PCR as described above.

#### Passive protection assay in suckling Kunming mice

To determine the potential protection effect of anti-JEVpr/DENV2 sera against DENV1–4 infection in vivo, the heat-inactivated anti-JEVpr/DENV2 sera or normal mouse sera were diluted at 1:10, and then incubated with equal volumes of DENV1–4 diluted in MEM medium (800 PFU) for 1 h at 37 °C, separately. The virus-serum mixtures were injected into groups of 3-day-old Kunming suckling mice by the ic route. Mice were then monitored for 15 days after inoculation.

### Statistical analysis

Statistical significance of differences between experimental groups was determined using the Student’s *t* test with SPSS 13.0 software. ANOVA Tukey’s post hoc test was used to compare the differences between the experimental groups and the control group for the ADE assay. A *p* value of less than 0.05 was considered significant.

## Results

### Construction and characterization of recovered DENV2

A full-length cDNA clone of DENV 2 strain ZS01/01 (plasmid pACYC177-DENV2) was linearized with *Cla*I prior to SP6 RNA polymerase transcription in the presence of a cap analog. The transcript showed one extra G residue at the 5′ end and an extra AU at the 3′ end when compared with the cDNA of DENV2. RNA transcripts were subsequently transfected into BHK-21 cells, and production of the progeny virus was monitored by immunofluorescence assay (IFA) using anti-E protein (4G2) antibodies. The number of infected cells increased with time, showing that the virus can be grown in cultured cells (data not shown). The recovered virus was then passaged several times in C6/36 cells, named RecDENV2. Full-genome sequencing showed that there were seven nucleotide substitutions between RecDENV2 and parental DENV2 and that three mutations in the E, NS2A, and NS5 genes were silent. The four amino acid mutations included E-192 Gly to Asp, NS2B-39 Gln to Pro, NS2B-114 Ile to Thr, and 2K-17 Val to Ala.

**Fig. 1:**
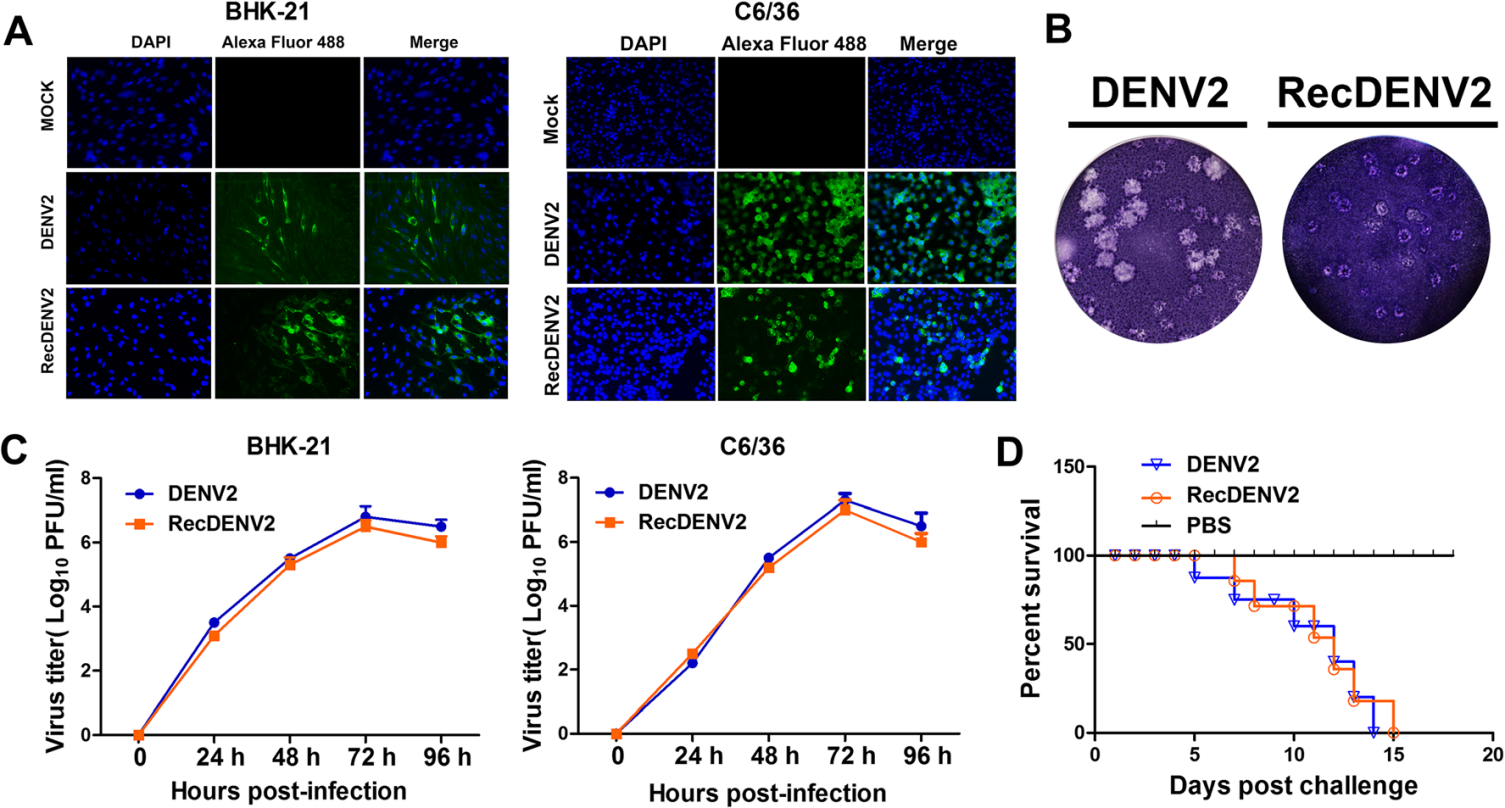
Characterization of RecDENV2. **a** IFA of RecDENV2, DENV2, or mock-infected BHK-21 and C6/36 cells with specific anti-E antibodies (4G2). **b** Plaque morphology of RecDENV2 and DENV2 on BHK-21 cells grown in 24-well plates were infected with a 10-fold serial dilution of the virus. The plates were incubated at 37 °C for 1 h. Supernatant was removed and cells were overlaid with 1.2 % carboxymethylcellulose in DMEM containing 2 % FBS. After further incubation in 37 °C for 7 days, the cells were fixed with 4 % formaldehyde and the plaques were visualized by staining with 0.8 % crystal violet. **c** Growth curves of the RecDENV2 and parental virus DENV2 in BHK-21 and C6/36 cells. Monolayers of BHK-21 and C6/36 cells were infected with RecDENV2 and parental virus DENV2 at an MOI of 0.01. At each point, the media were collected and virus titers were determined on BHK-21 cells by a plaque-forming assay. The means and standard errors of the means for virus titers were determined from three separate experiments. **d** Neurovirulence of the RecDENV2, parental virus DENV2, or PBS in suckling Km mice

Figure 1a shows the infectivity of RecDENV2 and parental DENV2 in C6/36 and BHK-21 cells detected by IFA with anti-E (4G2) antibodies. The recovered DENV2 caused cytopathic effects in BHK-21 cells similar to parental DENV2, and plaque assays revealed a slight reduction in plaque size in RecDENV2 (Fig. 1b). As shown in Fig. 1c, RecDENV2 and parental DENV2 were both replicated efficiently in C6/36 and BHK-21 cells and peaked at 72 h post-infection, with titers of 10^7.0^ and 10^7.3^ PFU/ml for C6/36 cells and titers of 10^6.5^ and 10^6.8^ PFU/ml for BHK-21 cells, respectively.

To investigate the neurovirulence properties of RecDENV2 and its parental DENV2, groups of 3-day-old Kunming mice were inoculated with 10-fold serial dilutions of RecDENV2 and parental DENV2 by the ic route. All of the infected mice were then observed for at least 21 days for signs of illness and death. All of the mice which received high inoculation dose (over 800 PFU/mouse) died within 7 days and all PBS controls survived for 15 days post-infection. Figure 1d shows the survival curve of mice immunized with 800 PFU RecDENV2 and parental DENV2. Compared to the parental DENV2, the time to death was delayed by 1 day in the mice from the RecDENV2 group. According to the result of 50 % lethal dose assay, the LD_50_ of RecDENV2 and its parental DENV2 were 10^−5^/20 μl and 10^–4.96^/20 μl, respectively. Taken together, these results indicate that the plaque phenotype, growth kinetics, and neurovirulence of recovered RecDENV2 were similar to those of parental DENV2.

### Engineering and characterization of chimeric JEVpr/DENV2

To study the effect of pr gene substitution on neutralization and antibody-dependent enhancement of dengue virus infection, we generated a chimeric JEVpr/DENV2 virus (JEVpr/DENV2) by replacing DENV2 pr with JEV pr based on the infectious cDNA clone of DENV2. The JEVpr/DENV2 virus was derived by transfection of in vitro-transcribed RNA into BHK-21 cells and was amplified in C6/36 cells. RT-PCR analysis and JEV pr gene sequencing confirmed that the pr gene of JEV was successfully engineered into the genome of the DENV2 cDNA clone as expected. The genome sequences aside from the pr gene between JEVpr/DENV2 and RecDENV2 were the same.

**Fig. 2:**
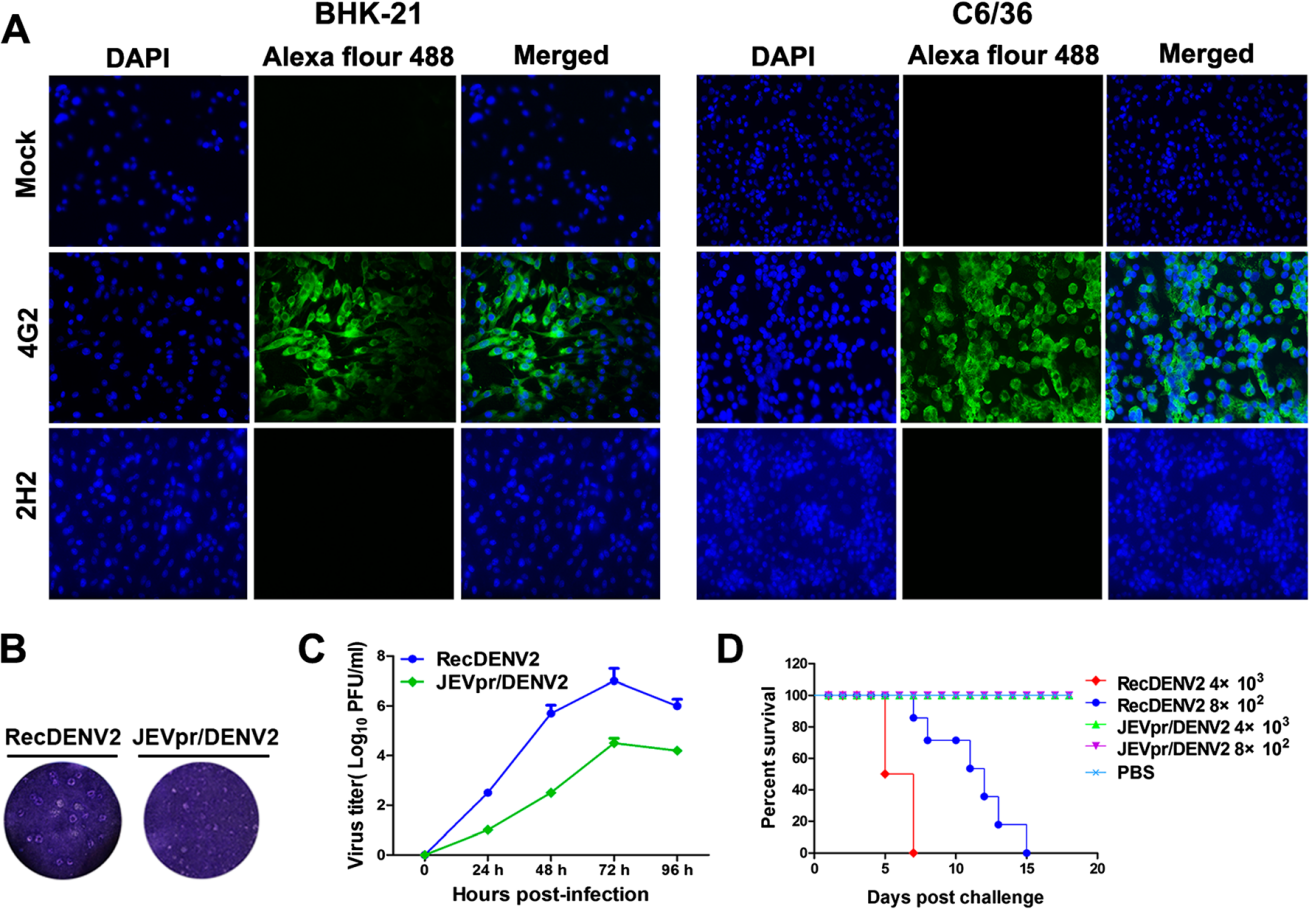
Characterization of JEVpr/DENV2. **a** IFA of JEVpr/DENV2- or mock-infected BHK-21 and C6/36 cells with specific anti-E antibodies (4G2) and anti-prM antibodies (2H2). Cells were infected with the chimeric JEVpr/DENV2 at an MOI of 0.01. At 48 h post-infection, normal cells and viruses were detected with specific anti-E antibodies (4G2) and anti-prM antibodies (2H2), respectively. **b** Plaque morphology of JEVpr/DENV2 and RecDENV2 on BHK-21 cells grown in 24-well plates were infected with a 10-fold serial dilution of the virus. The plates were incubated at 37 °C for 1 h. Supernatant was removed and cells were overlaid with 1.2 % carboxymethylcellulose in DMEM containing 2 % FBS. After further incubation in 37 °C for 7 days, the cells were fixed with 4 % formaldehyde and the plaques were visualized by staining with 0.8 % crystal violet. **c** Growth curves of the chimeric JEVpr/DENV2 and RecDENV2 in C6/36 cells. Monolayers of C6/36 cells were infected with the chimeric JEVpr/DENV2 or RecDENV2 at an MOI of 0.01. At each time point, the media were collected and virus titers in cell culture were determined on BHK-21 cells by plaque-forming assay. The means and standard errors of the means for virus titers were determined from three separate experiments. **d** Neurovirulence of the chimeric JEVpr/DENV2, RecDENV2, and PBS in suckling Km mice

Subsequently, IFA was performed to characterize the rescued JEVpr/DENV2 with specific anti-E antibodies (4G2) and anti-prM antibodies (2H2) (Fig. 2a). No fluorescence was detected when the cells were stained with primary antibody (2H2) due to the substitution of DENV pr peptide with JEV pr. The chimeric JEVpr/DENV2 caused typical cytopathic effects in BHK-21 cells and C6/36 cells similar to RecDENV2, producing a homogeneous population of plaques which were 0.25 mm in diameter, approximately one quarter of the size of those of RecDENV2 (Fig. 2b). The growth kinetics of chimeric JEVpr/DENV2 and RecDENV2 were subsequently determined in mosquito C6/36 cells. The results showed that JEVpr/DENV2 replicated less efficiently in C6/36 cells and peaked at 72 h post-infection, with a titer of 10^4.5^ PFU/ml, when compared with RecDENV2 (10^7.0^ PFU/ml) (Fig. 2c). Taken together, these results demonstrate that the chimeric JEVpr/DENV2 possesses the designed genomic structure and shows a consistent reduction of plaque size and titer.

### Neurovirulence of chimeric JEVpr/DENV2 in suckling mice

To compare the neurovirulence of chimeric JEVpr/DENV2 and RecDENV2, groups of 3-day-old Km mice (*n* = 8–12 mice/group) were inoculated (by ic route) with 4000 or 800 PFU RecDENV2 or JEVpr/DENV2 or PBS, respectively. Animals were monitored for 21 days after inoculation. As shown in Fig. 2d, the mice inoculated with RecDENV2 died within 7 (4000 PFU/mouse) and 15 days (800 PFU/mouse) post-infection with a lower body weight. The mice challenged with JEVpr/DENV2 (800 and 4000 PFU/mouse) all survived, with similar behavior to the control (PBS) group, without obvious weight loss.

### Immunogenicity of JEVpr/DENV2 and protection in adult BALB/c mice

**Fig. 3:**
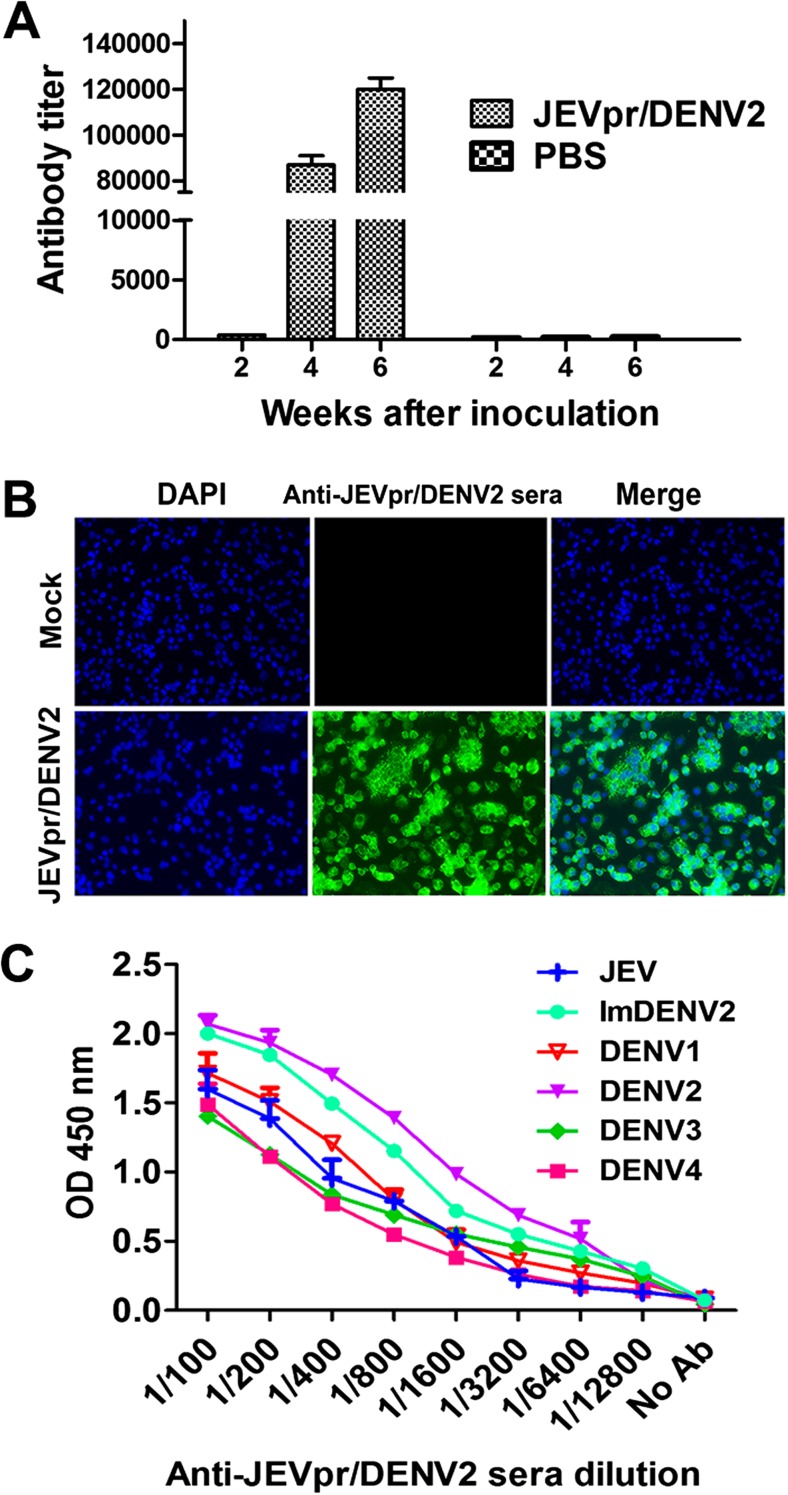
Immunogenicity and cross-reactivity of anti-JEVpr/DENV2 sera. Groups of 4-week-old BALB/c were inoculated with inactivated chimeric JEVpr/DENV2 or PBS by the sc route in the presence of Fruend’s adjuvant. Serum was collected from mice at 2, 4, and 6 weeks after the first inoculation for determination of titers of IgG antibody by ELISA. **a** Time course of antibody titer levels induced in mice immunized with JEVpr/DENV2 and PBS. **b** Specificity of anti-JEVpr/DENV2 sera with JEVpr/DENV2 detected by IFA. **c** Cross-reactivity of anti-JEVpr/DENV2 sera with DENV1–4, ImDENV2, and JEV measured by ELISA. Data are expressed as means of at least three independent experiments. The *error bars* represent standard deviations (SD)

To assess the immunogenicity and immunoreactivity of chimeric JEVpr/DENV2 in mice, serum antibody titers against JEVpr/DENV2 were measured by ELISA, where serum of 0 week at 1:100 dilution was used as the background. The antibody levels of mice immunized with three doses of inactivated JEVpr/DENV2 increased rapidly, and the antibody titer was remarkably higher than that of the PBS group (Fig. 3a). Furthermore, an indirect immunofluorescence assay was performed to detect the reactivity of anti-JEVpr/DENV2 sera with chimeric JEVpr/DENV2 (Fig. 3b). Then ELISA was applied to estimate the cross-reactivity of anti-JEVpr/DENV2 sera with natural viruses. The results showed that anti-JEVpr/DENV2 sera can cross-react with all four serotypes of DENV, ImDENV2, and JEV (Fig. 3c). These results indicated that JEVpr/DENV2 has good immunogenicity and the anti-JEVpr/DENV2 serum was highly cross-reactive.

### Protection response in adult BALB/c mice and Kunming suckling mice

**Fig. 4:**
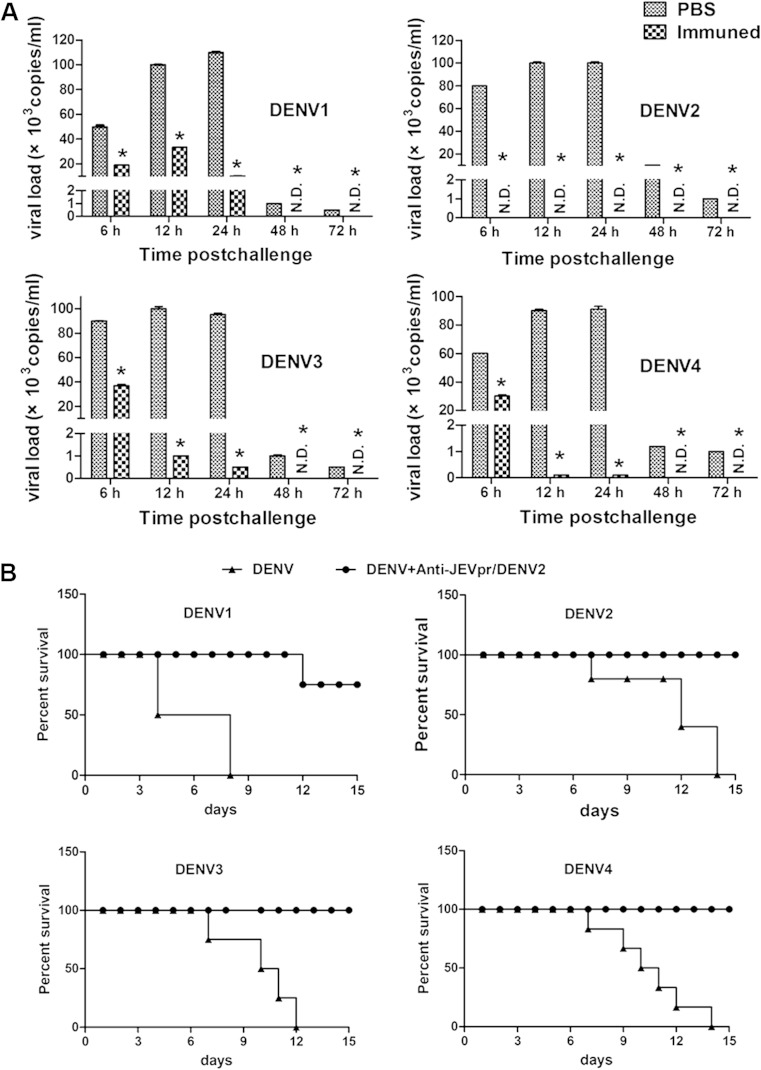
Protection test of anti-JEVpr/DENV2 sera. **a** Quantification of viral RNA levels in immunized mice after inoculated with DENV1–4. Two weeks after the last immunization with JEVpr/DENV2 or PBS, mice were infected with DENV1–4 by peritoneal injection. Viral RNA levels of sera were quantified by qRT-PCR at 6, 12, 24, 48, and 72 h post-infection. The viral RNA levels in the JEVpr/DENV2 group were lower than those of the PBS group at any given time point. **b** Protection test of mouse anti-JEVpr/DENV2 sera in Km suckling mice. The heat-inactivated anti-JEVpr/DENV2 sera or normal mouse sera were diluted at 1:10 and incubated with equal volumes of DENV1–4 diluted in MEM (800 PFU) for 1 h at 37 °C. The virus-serum mixtures were then injected into groups of 3-day-old Kunming suckling mice by the ic route. Data are expressed as means and SD of three independent experiments. The *error bars* represent standard deviations (SD). If there is no error bar, the variation was too small to show in the figure. *N.D.*not detectable; *significance (*P* < 0.05 vs PBS group) analyzed using Student’s *t* test

Two weeks after the last immunization, groups of mice were injected with DENV1–4 (10^6^ PFU/mouse) by the sc route. The viral RNA copy numbers of sera were quantified by qRT-PCR. High levels of viral RNA were detected at 6, 12, and 24 h post-infection in the PBS group, and the viral RNA copies were significantly reduced in all groups at 48, and 72 h post-challenge. However, the viral RNA levels in the mice immunized with JEVpr/DENV2 were significantly reduced or even undetectable after challenge with DENV (Fig. 4a). These results demonstrated that anti-JEVpr/DENV2 sera have some protection against DENV infection.

To further determine the protection of anti-JEVpr/DENV2 sera against DENV1–4 infection in suckling mice, DENV1–4 were incubated with equal volumes of anti-JEVpr/DENV2 sera or normal mouse sera at 1:10 dilution before being injected into groups of 3-day-old Kunming mice by the ic route, respectively. All of the mice challenged with DENV1–4 incubated with normal mouse sera died in 8, 14, 12, and 14 days post-infection. In the groups of mice challenged with DENV1–4 mixed with anti-JEVpr/DENV2 sera, except for a two mice death in DENV1 with anti-JEVpr/DENV2 group, the mice of the other three groups all survived past 15 days post-infection without obvious symptom (Fig. 4b). These results demonstrated that high concentrations of anti-JEVpr/DENV2 sera have a good neutralizing capacity to the four DENV serotypes and ImDENV2.

### Neutralization assay

**Fig. 5:**
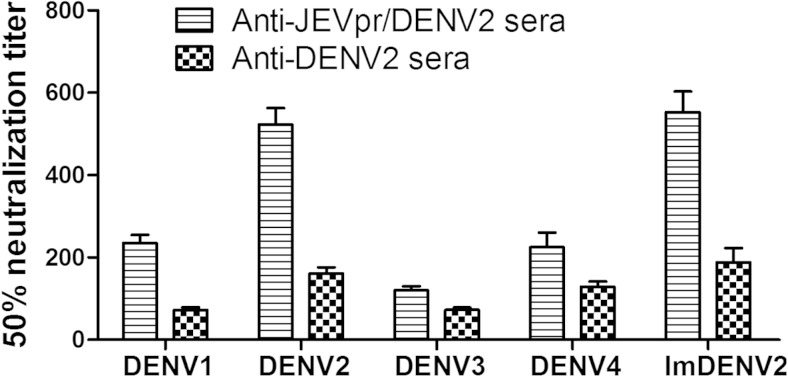
Neutralizing activity of anti-JEVpr/DENV2 sera and anti-DENV2 sera. Serial twofold dilutions of inactivated sera were used to calculate the 50 % neutralization titer against each virus. The mean 50 % neutralization titers are displayed in the graph. Data are expressed as means and SD of three independent experiments. The *error bars* represent standard deviations (SD). If there is no error bar, it is not that no variations among three independent experiments but that the variations are too small to show in the figure

A standard plaque reduction neutralization test (PRNT) was used to determine the neutralization titers of the anti-JEVpr/DENV2 sera and anti-DENV2 sera against DENV1–4 and ImDENV2 infection. Serial twofold dilutions of inactivated anti-JEVpr/DENV2 sera and anti-DENV2 sera (starting at 1:10) were mixed with approximately 30 PFU DENV1–4, JEVpr/DENV2, and ImDENV2 before testing by a plaque-forming assay. Then, the serum titer required to reduce dengue viral plaques by 50 % was calculated. As displayed in Fig. 5, with the anti-DENV2 sera, we obtained the mean 50 % neutralization titers of 72, 161, 73, 129, and 188 for DENV1, DENV2, DENV3, DENV4, and ImDENV2, respectively. For the anti-JEVpr/DENV2 sera, we calculated the mean 50 % neutralization titers of 235, 523, 120, 225, and 573 for DENV1, DENV2, DENV3, DENV4, and ImDENV2, respectively.

### Antibody-dependent enhancement of DENV infection

**Fig. 6:**
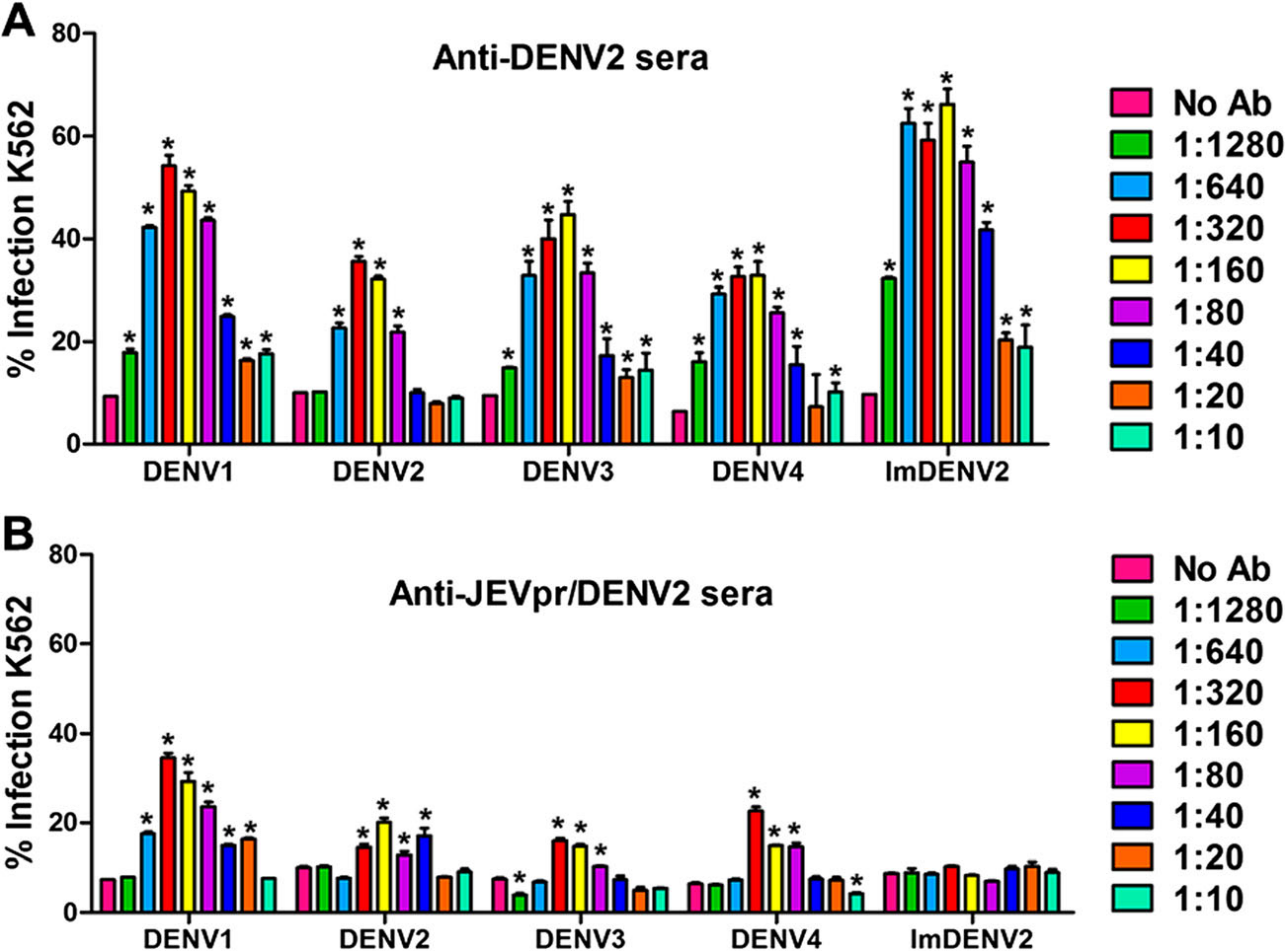
The enhancement of dengue virus infection by anti-DENV2 or anti-JEVpr/DENV2 sera in K562 cells. Serial twofold dilutions of antibody were incubated with an equal volume of DENV for 1 h at 37 °C before transferred to K562 cells at an MOI of 1. Percentage of infected K562 cells were determined by flow cytometry (**a** and **b**) with anti-E antibody (4G2) 3 days post-infection. Data are expressed as means of at least three independent experiments. The *error bars* represent standard deviations (SD). If there is no error bar, it is not that no variations among three independent experiments but that the variations are too small to show in the figure. *Significance (*P* < 0.05 vs no Ab group) analyzed using ANOVA Tukey’s post hoc tests

Next, we performed ADE assays using Fc receptor-bearing K562 cells to compare the anti-JEVpr/DENV2 sera and anti-DENV2 sera for their capacity to enhance DENV1–4 and ImDENV2 infection by flow cytometry. The results demonstrated that infection was enhanced in the two groups, where the percentage of enhanced infection percentage for anti-DENV2 sera varied from 7.01 to 44.87 % (DENV1), −2.11 to 25.52 % (DENV2), 3.5 to 35.24 % (DENV3), 0.86 to 26.49 % (DENV4), and 9.22 to 56.56 % (ImDENV2) (Fig. 6a). The percentage of enhanced infection for anti-JEVpr/DENV2 sera varied from 0.29 to 27.18 % (DENV1), −2.11 to 10.04 % (DENV2), −2.5 to 8.52 % (DENV3), and −2.16 to 16.24 % (DENV4) with a narrower range of serum concentrations. Surprisingly, anti-JEVpr/DENV2 sera had nearly no enhancing effect on ImDENV2 infection (−1.71 to 1.63 %) (Fig. 6b).

## Discussion

ADE has been thought to be an important cause of DHF/DSS, and the risk of ADE hampers the development of vaccines or specific antiviral therapies against DENV infection (Whitehead et al. 2007). Several investigations have reported that anti-prM antibodies have a significant role of enhancing DENV infection (Beltramello et al. 2010; Dejnirattisai et al. 2010; Huang et al. 2006; Rodenhuis-Zybert et al. 2010). Additionally, it has been demonstrated that the epitopes of these enhancing anti-prM antibodies were mainly located in the amino acid residues of the pr peptide (Dejnirattisai et al. 2010; Luo et al. 2015). A previous study also reported that there was relatively low sequence conservation between prM sequences (35 % DENV versus JEV), and only 3 % of the antibodies to dengue prM cross-react with JEV (Dejnirattisai et al. 2010). Therefore, we carried out this study to investigate the effect of pr gene substitution on neutralization and ADE of DENV infection by replacing the pr gene of DENV with that of JEV.

Reverse genetics technology is extensively used to generate recombinant DENV which are modified by deletion, or by antigenic chimerization between two related viruses to study the biological characteristic changes, and thus further study the structure and function of the virus genome as well as the pathogenesis of DENV infection and the development of DENV vaccine candidates (Keelapang et al. 2004; Wahala et al. 2012). Reverse genetics has proven to be a very valuable tool to introduce defined attenuating mutations into DENV genomic RNA. In this study, we first constructed the full-length cDNA clone of DENV2 (pACYC177-DENV2) utilizing reverse genetics. A comparison of the 5′ and 3′ terminal sequences revealed that both RNA transcripts and RecDENV2 have one extra G residue at the 5′ end, and this extra G residue has no obvious effect on the infectivity of the recovered virus. This result was comparable to what has been reported for other *flavivirus* infectious transcripts (Polo et al. 1997; Yun et al. 2003). The RNA transcripts of DENV2 had an extra AU at the 3′ end while RecDENV2 did not. We suspect that the two extra nucleotides do not remain a part of the transcript-derived RecDENV2, and this phenomenon was also observed in another study previously reported (Khromykh and Westaway 1994).

The similar infectivity, plaque phenotype, and neurovirulence between RecDENV2 and DENV2 indicated that this method was practicable. Next, a chimeric JEVpr/DENV2 was constructed by replacing the DENV pr gene with that of JEV based on pACYC177-DENV2. The chimeric JEVpr/DENV2 displayed significantly reduced infectivity, plaque size, and mouse neurovirulence compared to RecDENV2 (Fig. 2a–c). These results are consistent with previous observations and indicate that the reduction in infectivity and plaque size for JEVpr/DENV2 is likely to be determined partly by the virus structural protein genes (Chambers et al. 1999).

Three doses of JEVpr/DENV2 stimulated efficient immune responses in mice, and the antibody against JEVpr/DENV2 increased rapidly after each sequential immunization. These results demonstrate that the chimeric JEVpr/DENV2 have good immunogenicity (Fig. 3). Moreover, we found that anti-JEVpr/DENV2 lead to a significantly reduced viral load in the mice during an in vivo protection assay. A previous study showed that high levels of plasma dengue viral load are related to disease severity (Wang et al. 2003). Our data indicated that JEVpr/DENV2 indeed elicited protective antibodies against DENV. Without a doubt, virulence attenuation and the protective effect of JEVpr/DENV2 need to be further investigated in non-human primates. In addition, recent studies have suggested that an AG129 mouse model would be an appropriate animal model to investigate the neutralization and ADE of DENV infection in vivo (Balsitis et al. 2010; de Alwis et al. 2014; Fuchs et al. 2014).

Antibodies induced by DENV may have dual roles: obstruct infection through neutralization activity or enhance infection by ADE. In agreement with the results of several previous studies (Beltramello et al. 2010; de Alwis et al. 2014), both anti-JEVpr/DENV2 sera and anti-DENV2 sera showed broad cross-reactivity with all four DENV serotypes. Meanwhile, anti-JEVpr/DENV2 sera have a good neutralizing capacity to the four DENV serotypes and ImDENV2 in vitro and in vivo. The 50 % neutralization titers of the anti-JEVpr/DENV2 sera were higher than that of anti-DENV2 sera. These results indicated that anti-prM antibodies were not the main target of human neutralizing antibodies, and this result is consistent with previous studies (Beltramello et al. 2010; Dejnirattisai et al. 2010; Luo et al. 2013). The partial cleavage of prM reduces available antigens for neutralization. This, together with the cross-reactivity among the four DENV serotypes, leads to the susceptibility of DENV to ADE mediated by prM antibodies (Rodenhuis-Zybert et al. 2011b). A recent study reported that anti-prM antibodies could render essentially non-infectious immature particles highly infectious (Rodenhuis-Zybert et al. 2010). Therefore, we set out to reduce or eliminate the ADE by replacing DENV pr with JEV pr. We compared the enhancing activity of anti-JEVpr/DENV2 sera and anti-DENV2 sera to analyze the role of pr antibodies in ADE. The results showed that anti-DENV2 sera were highly cross-reactive and showed potent ADE activity over a broad range of concentrations. However, anti-JEVpr/DENV2 sera showed a significant decrease in the percentage of infection enhancement. Meanwhile, the range of anti-JEVpr/DENV2 sera concentrations with different enhancing activities was also narrowed. Because only 3 % of the antibodies to prM cross-reacted with JEV (Dejnirattisai et al. 2010), the anti-prM responses were minimized. Our results reported here indicate that the ADE mediated by anti-pr antibodies could be reduced by replacing the pr gene with JEV pr at the whole genome level. The enhancing capacity of anti-JEVpr/DENV2 sera was not eliminated completely mainly due to the existence of enhancing epitopes on the E or NS1 protein (da Silva Voorham et al. 2012; Masrinoul et al. 2013; Rodenhuis-Zybert et al. 2011a). In addition, another study has shown that the anti-E monoclonal antibodies could react with peptides from the prM protein (Falconar 1999). Strikingly, anti-JEVpr/DENV2 sera had no detectable enhancing activity of ImDENV2 infection in the present study. This may be because the concentration of infectious antibody-virus complexes is not great enough to elicit the systematic response and the infection enhancement is gradually lost (Halstead 2003).

Most current DENV vaccine candidates, including naturally attenuated, recombinantly attenuated, chemically inactivated viruses, and DENV-yellow fever chimeras, contain native dengue prM sequences (Murphy and Whitehead 2011; Sjatha et al. 2014). It may be worthwhile to consider alternative vaccine approaches that minimize or eliminate anti-prM responses during vaccine design. Vaccine candidates that reduce or eliminate the ADE of DENV infection may be increasingly important. In this study, we characterized a chimeric virus JEVpr/DENV2, which showed reduced virulence and good immunogenicity. Also, anti-JEVpr/DENV2 sera showed broad cross-reactivity and efficient neutralizing activity with all four DENV serotypes and immature DENV2 (ImDENV2) in vitro and in vivo. Most importantly, anti-JEVpr/DENV2 sera showed significantly reduced enhancing activity of DENV infection in K562 cells as compared with anti-DENV2 sera. These results suggested that the infection-enhancing activities could be reduced by replacing the DENV pr gene with JEV pr gene. These findings may provide evidence for better understanding of the role of anti-prM antibodies in the pathogenesis of DENV infection and for the development of DENV vaccines.
